# High-Throughput NIR-Chemometric Method for Meloxicam Assay from Powder Blends for Tableting

**DOI:** 10.3797/scipharm.1108-07

**Published:** 2011-10-13

**Authors:** Ioan Tomuta, Rares Iovanov, Andreea Loredana Vonica, Sorin E. Leucuta

**Affiliations:** 1 Department of Pharmaceutical Technology and Biopharmaceutics, “Iuliu Hatieganu” University of Medicine and Pharmacy, 41 V. Babes Street, 400023, Cluj-Napoca, Romania; 2 S. C. Polipharma Industries, 550052, Sibiu, Romania

**Keywords:** Near Infrared Spectroscopy, Chemometrics, Meloxicam assay, Powder blends, Method validation

## Abstract

A near infrared (NIR) method able to directly quantify the active content in pharmaceutical powder blends used for manufacturing meloxicam tablets, without any sample preparation, was developed and fully validated. To develop calibration models for the assay of meloxicam in powder blends for tableting, the NIR reflectance spectra of different meloxicam powder blends prepared according to a calibration protocol was analysed using different preprocessing methods by partial last-square regression (PLS) and principal component regression (PCR).

The best calibration model was found when partial last-square regression (PLS) was used as regression algorithm in association with Smoothing-Savitsky as pre-processing spectrum method. The trueness, precision (repeatability and intermediate precision), accuracy, linearity and range of application of the developed NIR method were validated according to the International Conference of Harmonization (ICH) and Medicine European Agency (EMA) guidelines and found to be fit for its intended purpose.

## Introduction

The concept of Process Analytical Technology (PAT) was introduced by FDA, EMA and ICH in drug manufacturing guidance [1–3]. Near Infrared (NIR) spectroscopy is an important component of a PAT toolbox and is a key technology for enabling rapid analysis of the manufacturing process of pharmaceutical tablets. Direct analysis of pharmaceutical powder blends or intact solid dosage forms is considered to be a very important goal for NIR Attributes (CQAs) as an analysis of pharmaceutical solids, with the increasing needs for on-line or at-line testing [4, 5].

As a PAT tool, Near Infrared (NIR) spectroscopy is extensively used to monitor such Critical Quality moisture content in the granules and crystal forms of a drug during the granulating-drying process [6, 7], compact hardness during the roller compaction process [8] and blend uniformity during the powder blending process [9, 10] because of its rapid and non-destructive process. The NIR spectrum of a powder sample reflects both chemical and physical information [11, 12].

Thus, NIR spectroscopy can be used to perform quantitative determinations of specific compounds in complex matrices, like, for instance, a pharmaceutical powder blend for tableting. A NIR assay method is usually not performed in the “traditional” way like UVspectrometry or HPLC. The major step to develop a NIR assay method is the calibration procedure for model development. Once a calibration is developed and favourable predictions are expected, they must be validated to be accepted for routine use. For external validation, independent sets of samples are needed. There are several validation parameters that must be determined in order to be consistent with the recommendations of the International Conference of Harmonisation (ICH) and with other guidelines: accuracy, precision (repeatability and intermediate precision), specificity, linearity and range of application [10, 11, 13].

Ensuring an appropriate calibration concentration range is one of the main limitations of NIR spectroscopy applications in quantitative applications in the pharmaceutical industrial environment. Concentration variability is necessary to guarantee the method’s robustness. A desirable range between 80% and 120% would be advantageous to improve the accuracy of the calibration. Several methods were proposed to set up a NIR-based calibration method:

- The use of normal production tablets with development of samples which would normally be out of specification, in order to extend the concentration range.
- The use of laboratory-made samples, developed by changing the concentration of the components in the matrix.
- The use of laboratory samples with production samples, comprising granules, tablet cores and coated tablets, in order to include all sources of variation in the model [11–15].

The most frequent methods to construct the calibration set are the use of doped production samples (method of standard addition) and the use of laboratory-made samples. A number of papers compare the methods and conclude that both methods produce similar results [11, 12].

The aim of this work was to develop and validate a NIR-chemometric method for the assay of meloxicam in powder blends for tableting, based on laboratory made samples, in order to be used for in-line/at-line meloxicam pharmaceutical process monitoring.

## Results and Discussion

The aim of our research was to develop a NIR-chemometric method suitable for the direct quantification of meloxicam in powder blends for tableting, without any sample preparation. To ensure appropriate quantitative results, the set of calibration samples has to be representative of the changes in the properties of samples that will be found in routine analysis. In general, the calibration set will contain an approximately balanced distribution of samples across the concentration domain and a considerable number of representative samples for each level of concentration of the substance of interest. The matrix of the calibration protocol was presented in Table 2.

### Method development

**Fig. 1:**
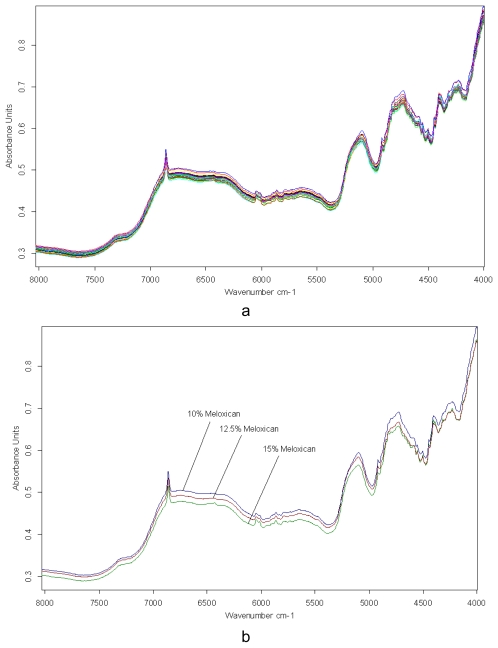
Reflectance spectrum of all calibration powder blends (a) and of the powder blends at three concentration levels (10%, 12.5%, 15%) of meloxicam (b)

Spectra investigation. The development of a calibration model consisted in checking different spectral pre-treatments as well as their combination with different spectral ranges. Both the whole spectral range and specific spectral regions containing strong bands and different spectral pre-treatments were tested with a view to constructing the calibration models. The near infrared spectra of the calibration powder blends are shown in figure 1a. The spectra of the three fractions used in the calibration model are shown in Figure 1b. As shown in figure 1b, significant differences are present especially in the 7500–4000 cm^−1^ range of the spectrums. This region was used for model calibration.

To make quantitative analysis using NIR spectroscopy, mathematical and statistical methods (chemometric methods and multivariate analysis) are required, which will extract the relevant information and reduce the irrelevant information. Spectral interference parameters are calling for mathematical corrections, the so-called spectra pre-treatments, to reduce, eliminate or standardize their impact on the spectra.

In this study, the model development consisted in checking different spectra pre-treatments in combination with the whole spectra or different spectral regions containing strong bands of meloxicam. Multivariate calibration was then applied to chemometric approaches based on PLS and PCR regression. After designing the calibration model, its predictive ability was tested with the samples used during its development. The validation of the model was done using the cross-validation method, leaving out one sample at a time, and the predicted concentrations were compared with the known concentrations of the compounds in each sample. The RMSECV was used as a diagnostic test for examining the errors in the predicted concentrations because it indicates both precision and accuracy of predictions. It was calculated upon addition of each new factor to the PLS and PCR models. For each pre-processing method, the squared correlation coefficient, R^2^, between actual known concentration and predicted concentration, was computed to evaluate the predictive ability of the model.

The optimal number of factors was selected by the following criteria: the selected model is that with the smallest number of factors such that RMSECV for that model is not significantly greater than RMSECV for the model with one or more additional factors [14].

**Fig. 2:**
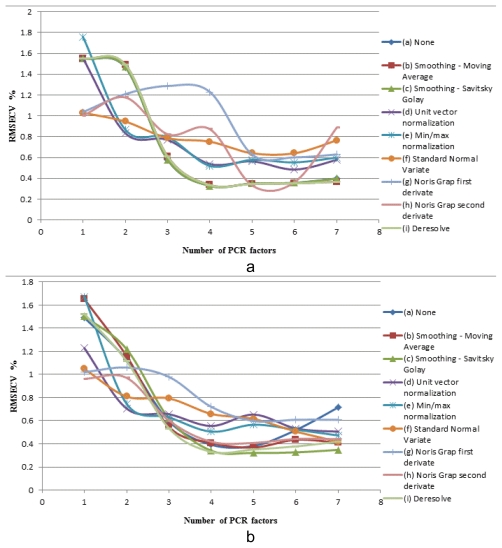
Plotting RMSECV vs. (a) number of PCR factors, (b) number of PLS factors

**Fig. 3:**
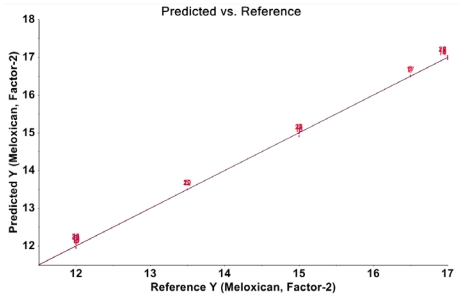
NIR predicted meloxicam concentration versus true meloxicam concentration, cross-validation

The NIR-chemometric model was developed taking into account all the samples of the three series of samples from the calibration matrix. The results obtained during the method development are presented in Tables 4 and 5 and Figures 2 and 3.

Concerning the results, the R^2^ values for the proposed models were greater than 0.98, in (b), (c), (h) and (i) PCR models and (c) and (i) PLS models. The lowest number of PCR factors was 4 for models (b), (c) and (i), and the lowest number of PLS factors was also 3 for models (b), (c), (d) and (1). Considering the RMSECV and bias for those models, together with the R^2^ values and the number of factors, the (c) model, using the Smoothing-Savitsky method, has been chosen as the best fitted model for meloxicam quantification in powder blends for tableting, using the PLS algorithm.

Figure 2 shows the RMSECV plotted as a function of (a) PCR and (b) PLS factors for the quantification of meloxicam in powder blends for tableting with different spectra preprocessing methods.

Based on the results presented in Table 4, 5 and Figure 2, the Smoothing - Savitsky pre-treatment has been chosen as the best fitted model for meloxicam quantification, using the PLS algorithm and the optimal number of factors as 4.

The plots of predicted concentration versus true concentration for meloxicam in powder blends for tableting are shown in Figure 3. The predicted values were obtained using the Smoothing-Savitsky pre-treatment method, PLS algorithm and 4 PLS factors chosen in the calibration of model.

### Method validation

The model predictive performance was evaluated with accuracy profiles computed on the external validation results. This approach is using tolerance intervals as statistical methodology that allows predicting a region of concentration where each future result has a probability to fall defined by the analyst [19]. The accuracy profile has the advantage of taking into account the total error, which is the sum of the trueness (systematic error) and precision (random error), and meets the ICH Q2 (R1) guideline requirements [20–22].

Table 7 shows the ICH Q2 (R1) validation criteria of the developed method. The trueness of the method was evaluated by calculating the recovery. The recovery has satisfactory values for all three concentration levels (Table 6). The precision of the method was evaluated by calculating two parameters: repeatability and intermediate precision at the three concentration levels in the validation protocol. Both parameters had satisfactory values for all concentration levels (Table 6).

The linear profile of the prediction model is shown in Table 6 and Figure 5. The linear model was represented by plotting the calculated concentrations of the validation samples as a function of the introduced concentrations. The dashed limits on the graph correspond to the accuracy profile and the dotted curves represent the acceptance limits at ±15% expressed in the concentration unit. As seen in the figure, the R^2^ value is 0.9956 and the slope is very close to 1, confirming the linearity of the model for quantification of meloxicam in powder blends for tableting.

**Fig. 4:**
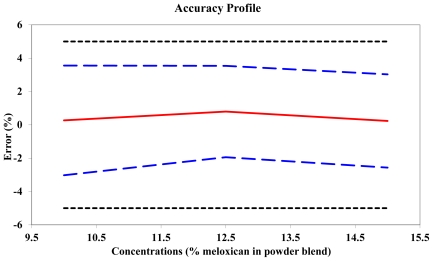
Accuracy profile of the NIR method for the quantification of meloxicam in powder blends

**Fig. 5:**
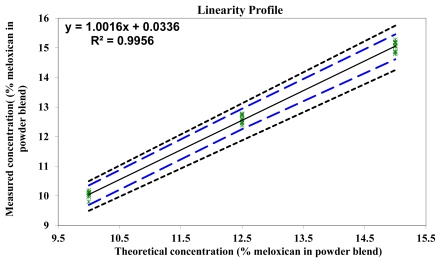
Linearity profile of the NIR method for the quantification of meloxicam in powder blends

As shown in Table 6 and Figure 4, the accuracy of the method for the entire meloxicam concentration domain is good, because the β-expectation tolerance limits never exceed the ±15% acceptance limits. The best accuracy was obtained at the concentration level of 12.5 % meloxicam in powder blend for tableting.

## Conclusions

A near infrared method was developed for the quantification of meloxicam in powder blends for tableting. Different calibration models were evaluated for the quantification of meloxicam in powder blends for tableting, ranging from 10–15%, using NIR-chemometic technique. Then, using the best calibration model, the method was fully validated according to the ICH guidance. The validation results showed good precision, trueness and accuracy for the determination of meloxicam in powder blends for tableting with contents ranging from 10–15% meloxicam in powder blend (coresponding to 80–120% active substance content).

The proposed NIR method allows the active substance (meloxicam) content in pharmaceutical powder blends for tablet manufacturing to be determined with no sample preparation. In another research paper [24] the authors have shown that it is possible to directly predict from the NIR reflection spectrum the pharmaceutical properties of the same meloxicam powder blends, such as mean particle size, poly-dispersion index, angle of repose and the time of flow, without any sample preparation.

Such quick NIR-chemometric methods can be used for on-line, in line or at line monitoring of the manufacturing process of meloxicam tablets and are helpful in achieving the goals of the process analytical technology (PAT) concept.

## Experimental

### Materials

Meloxicam (Uquifa, Spain), isomalt (BENEO-Palatinit GmbH, Germany), microcrystalline cellulose (JRS Pharma, Germany), sodium starch glycolate (JRS Pharma, Germany), silicon dioxide–Aerosil (RohmPharma Polymers, Germany), magnesium stearate (Union Derivan, Spain).

### Preparation of powder blends for NIR calibration

The formula of the powder blends for the preparation of meloxicam tablets are presented in Table 1.

In detail, meloxicam, isomalt, microcrystalline cellulose, and sodium starch glycolate were mixed using a planetary mixer (PRS type, Erweka, Germany) for 5 min. Magnesium stearate was added; the mixing was continued for 1 more minute.

### Calibration and validation protocol

An experimental protocol was followed for the calibration and validation steps in order to develop and to validate a robust near infrared model. This protocol included batches and days as sources of variability for the calibration as well as for the validation steps. Five concentration levels of active pharmaceutical ingredient (meloxicam) were evaluated (Table 2).

### Calibration samples

Laboratory batches of meloxicam powder blends for tablet preparation were manufactured according to the method previously described. The usual targeted formulation contains 12.5% w/w meloxicam (15 mg/tablet). This formulation will be further considered as the 100% active content formulation. For the calibration set, five laboratory batches containing 10, 11.25, 12.50, 13.75 and 15%w/w meloxicam were also manufactured (corresponding to 80, 90, 100, 110 and 120% API formulations). The amount of microcrystalline cellulose was adjusted to give a 120 mg final weight for each tablet, as shown in Table 3. Three independent batch series of five concentration levels were manufactured.

### External validation samples

New laboratory batches of meloxicam powder blends for tablets were manufactured for validation. The same formulations as for the calibration samples were manufactured at three concentration levels (levels 1, 3 and 5 from Table 3), corresponding to the 80, 100 and 120% concentrations of the API formulations. Four independent batch series were manufactured for each concentration level.

### NIR-spectroscopic analysis

The powder blend samples were analysed using a NIR model Antaris II FT-NIR Analyser (TermoElectron, SUA) in Reflectance Sampling configuration. Each reflectance spectrum was recorded using OMNIC software by integrating 32 scans taken from 11000 to 4000cm^−1^ at 8cm^−1^ resolution.

### Model development

#### Calibration and prediction

The model with the biggest predictive potential was selected according to conventional classical criteria such as the R^2^, the number of PLS factors, the Root Mean Square Error of Calibration (RMSEC) and the Root Mean Square Error of Estimation (RMSEE) [16]. Those parameters were calculated by the Unscrambler software package (Camo, Norway). The software allows validating the models by full cross-validation. In this procedure, iterative calibrations were performed removing in turn each standard from the training set and then predicting the excluded sample with that calibration.

#### Spectra preprocessing and regression methods

The model was developed with various combinations of the mathematical *pre-processing*. The spectral pre-treatments tested with a view to constructing the calibration models included the first and second derivative, and the Standard Normal Variate (SNV). First and second spectral derivatives were obtained by applying the Savitzky–Golay algorithm to 11 moving window points and a second-order polynomial. The influence of two standard regression methods, principal component regression (PCR) and partial least squares (PLS) regression, were also examined, selected because they were the most accurate in terms of model complexity and the number of samples used to build the calibrations. The optimal numbers of factors for PCR and PLS were determined by a cross-validation procedure with groups of two spectra (each side of the sample being represented by a spectrum) [17, 18].

### Method validation

Once a calibration is developed and favourable predictions are expected, they must be validated to be accepted for routine use. Independent sets of samples are needed for external validation. There are several validation parameters that must be determined in order to be consistent with the recommendations of the International Conference of Harmonization (ICH) and with other guidelines: accuracy, precision (repeatability and intermediate precision), linearity and range of application. The validation was performed according to the strategy proposed by Hubert et al [20–23].

## Figures and Tables

**Tab. 1:** Qualitative and quantitative formula of powder blends for tablet preparation

	mg / tablet	%
Meloxicam	15.00	12.50
Isomalt	52.20	43.50
Microcrystalline Cellulose PH 102	45	37.50
Sodium starch glycolate	6	5.00
Silicon dioxide	1.2	1.00
Magnesium stearate	0.6	0.50

	120.00	

**Tab. 2:** Matrix of calibration and validation protocol

Concentration level		Series 1		Series 2		Series 3	
		Calibrat.	Validat.	Calibrat.	Validat.	Calibrat.	Validat.
1	80%	1	4	1	4	1	4
2	90%	1	0	1	0	1	0
3	100%	1	4	1	4	1	4
4	110%	1	0	1	0	1	0
5	120%	1	4	1	4	1	4

**Tab. 3:** Composition of calibration samples and external validation samples

Concentration Levels	1^a^,^b^	2^a^	3^a^,^b^	4^a^	5^a^,^b^
	80%	90%	100%	110%	120%
Meloxicam (w/w)	10.00%	11.25%	12.50%	13.75%	15.00%

**Tablet composition (mg/tablet)**					

Meloxicam	12	13.5	15	16.5	18
Isomalt	55.2	53.7	52.2	50.7	49.2
Microcrystalline Cellulose	45	45	45	45	45
Sodium starch glycolate	6	6	6	6	6
Silicon dioxide	1.2	1.2	1.2	1.2	1.2
Magnesium stearate	0.6	0.6	0.6	0.6	0.6

	**120**	**120**	**120**	**120**	**120**

a calibration samples;

b external validation samples.

**Tab. 4:** Statistical parameters and numbers of principal components in the PCR method, without data pre-treatments, as well as after different spectra pre-treatments

Pre-treatment	Model	PC number	RMSECV	RMSEP	R^2^	Bias
(a) None	PCR	6	0.3274	0,3571	0.9740	0.0250
(b) Smoothing-Moving Average	PCR	4	0.3356	0.3554	0.9831	0.0254
(c) Smoothing-SavitskyGolay	PCR	4	0.3270	0.3424	0.9841	0.0250
(d) Unit Vector Normalization	PCR	5	0.5368	0.5606	0.9569	0.0347
(e) Min/max normalization	PCR	5	0.5194	0.5721	0.9593	0.0293
(f) Standard Normal Variate	PCR	5	0.6407	0.6671	0.9398	0.0298
(g) NorisGrap first derivate	PCR	5	0.6279	0.6546	0.9408	0.0352
(h) NorisGrap second derivate	PCR	5	0.3306	0.3622	0.9836	0.0045
(i) Deresolve	PCR	4	0.3348	0.3504	0.9832	0.0252

**Tab. 5:** Statistical parameters and numbers of the principal components in the PLS method, without data pre-treatments, as well as after different spectra pre-treatments

Pre-treatment	Model	PC number	RMSECV	RMSEP	R^2^	Bias
(a) None	PLS	5	0.39233	0.3966	0.9789	0.0707
(b) Smoothing-Moving Aveage	PLS	4	0.40885	0.4702	0.9768	0.1127
(c) Smoothing-SavitskyGolay	PLS	4	0.32453	0.3316	0.9889	0.0501
(d) Unit Vector Normalization	PLS	4	0.55465	0.5755	0.9543	−0.0115
(e) Min/max normalization	PLS	5	0.50516	0.5542	0.9616	0.0348
(f) Standard Normal Variate	PLS	7	0.41884	0.4501	0.9744	0.0670
(g) NorisGrap first derivate	PLS	6	0.59724	0.6202	0.9483	0.0121
(h) NorisGrap second derivate	PLS	5	0.41767	0.4455	0.9742	0.0572
(i) Deresolve	PLS	4	0.33095	0.3462	0.9826	0.0243

**Tab. 6:** Validation results of the NIR method for meloxicam quantification in powder blends

**Trueness**	**Concentration level (% meloxicam)**	**Mean concentration (% meloxicam)**	**Recovery (%)**		
	10.00	10.03	100.27		
	12.50	12.60	100.80		
	15.00	15.03	100.23		

**Precision**	**Concentration level (% meloxicam)**	**Relative bias (%)**	**Repeatability (RSD %)**	**Intermediate precision (RSD %)**	
	10.00	0.2673	1.359	1.183	
	12.50	0.7985	1.116	0.982	
	15.00	0.2320	1.158	1.006	

**Accuracy profile**	**Concentration level (% meloxicam)**	**Lower RelTol. Limit (%)**	**Upper RelTol. Limit (%)**	**Lower acceptance limit (%)**	**Upper acceptance limit (%)**
	10.00	−3.022	3.557	−5	5
	12.50	−1.943	3.539	−5	5
	15.00	−2.564	3.028	−5	5

**Linearity Profile**	**Concentration**	**Lower Tol. Limit (% meloxicam)**	**Upper Tol. Limit (% meloxicam)**	**Lower acceptance limit (% meloxicam)**	**Upper acceptance limit (% meloxicam)**
	10	9.696	10.356	9.500	10.500
	12.5	12.254	12.945	11.875	13.125
	15	14.614	15.455	14.250	15.750
